# Magnetoelectric nanoparticles shape modulates their electrical output

**DOI:** 10.3389/fbioe.2023.1219777

**Published:** 2023-08-25

**Authors:** A. Marrella, G. Suarato, S. Fiocchi, E. Chiaramello, M. Bonato, M. Parazzini, P. Ravazzani

**Affiliations:** Cnr-Istituto di Elettronica e di Ingegneria dell’Informazione e delle Telecomunicazioni, Milano, Italy

**Keywords:** magnetoelectric nanoparticles, nervous system stimulation, electroporation, piezoelectric materials, ferromagnetic materials, core-shell structures, wireless stimulation

## Abstract

Core-shell magnetoelectric nanoparticles (MENPs) have recently gained popularity thanks to their capability in inducing a local electric polarization upon an applied magnetic field and *vice versa*. This work estimates the magnetoelectrical behavior, in terms of magnetoelectric coupling coefficient (αME), via finite element analysis of MENPs with different shapes under either static (DC bias) and time-variant (AC bias) external magnetic fields. With this approach, the dependence of the magnetoelectrical performance on the MENPs geometrical features can be directly derived. Results show that MENPs with a more elongated morphology exhibits a superior αME if compared with spherical nanoparticles of similar volume, under both stimulation conditions analyzed. This response is due to the presence of a larger surface area at the interface between the magnetostrictive core and piezoelectric shell, and to the MENP geometrical orientation along the direction of the magnetic field. These findings pave a new way for the design of novel high-aspect ratio magnetic nanostructures with an improved magnetoelectric behaviour.

## 1 Introduction

Magnetoelectric nanomaterials have attracted a great interest in the last years in different fields of nanotechnologies and biomedical research, due to their capability of exhibiting at room temperature the so called “magnetoelectric” (ME) effect, which is a linear coupling between an external magnetic field and a generated, local polarization (and *vice versa*) (Kopyl et al., 2021; Smith et al., 2022; Song et al., 2022). In particular, magnetoelectric structures are composed by a 1) magnetostrictive phase, which experiences a non-zero strain when subjected to an external magnetic field and a 2) piezoelectric phase, which transduces the mechanical deformation absorbed into electric charges (Betal et al., 2016b). This class of materials constitutes an enormous asset to locally induce electric charges within the human body, which can be triggered by relatively low external magnetic field, without neither causing substantial mechanical stress in the surroundings nor requiring invasive wire connections.

The potential of ME nanostructures to provide a wireless stimulation of specific cells, organelles and tissues opens terrific possibilities in the field of biotechnologies. It is in fact widely recognized that a plethora of biological mechanisms, such as tissue growth and regeneration (Mushtaq et al., 2019; Zhang et al., 2021), cellular apoptosis and protein secretion, are modulated by electric stimuli. In addition, numerous biological structures (i.e., cellular membranes, intra- and extra-cellular microenvironments) base their functioning on a precise exchange and balance of ions and free electrical charges for a healthy homeostasis (Smith et al., 2022). If any alteration of these physiological electrical states occurs, it would be of major help to rely on a technological nanotool to both sense the electrical shift and restore it. Under this framework, designing a new generation of materials able to finely tune their electric behavior with a high spatial resolution and in a complete non-invasive way (i.e., avoiding the use of implanted, wired electrodes), either through external magnetic stimulation or by exploiting their piezoelectric nature is gaining a tremendous interest (Fernandes et al., 2019).

Currently, ME nanoparticles decorated with bioactive compounds have been used for different applications, such as on-demand drug delivery (Abdelazim, 2017; Mhambi et al., 2021; Shahzad et al., 2021; Casillas-Popova et al., 2022; Song et al., 2022), nano-electroporation (Kaushik et al., 2017), and brain stimulation (Yue et al., 2012; Kozielski et al., 2021). More specifically, numerical simulation studies (Guduru et al., 2018; Bok et al., 2022) have highlighted that ME materials can operate as enablers for a detailed mapping of the brain, since they generate a magnetic moment when in proximity with a neuron firing its action potential, thus performing a “sensing-type” of read out (“inverse” magnetoelectric effect). As a complementary, yet paramount function, ME particles have been proposed as nanoelectrodes for wireless brain stimulation (Yue et al., 2012; Fiocchi et al., 2022a; Chiaramello et al., 2022), and studied onto embryonic hippocampal cells *in vitro* (E. Zhang et al., 2022), delivered to cortical slices *ex vivo* (Nguyen et al., 2021), or administrated via intranasal route (Pardo et al., 2021) or via injection at the subthalamic region (Kozielski et al., 2021) to remotely modulate neuronal response and wirelessly tailor the local brain activity in a mouse model. In this context, single-neuron spatial resolution coupled with minimal energy dissipation achievable with MENPs, as well as the remote activation and control by means of a safe magnetic source, are of great interest for the treatment of various neuropsychiatric disorders. Cutting-edge nanomaterials have emerged and investigated to act as nano-transducers in order to modulate brain activity through optogenetic (Yizhar et al., 2011; Kim et al., 2017; Chen et al., 2021), mechanical (Yu et al., 2022), magneto-thermal (R. Chen et al., 2015; Hescham et al., 2021; Moon et al., 2020; Rosenfeld et al., 2022; Wu et al., 2021), electrical strategies (Li et al., 2021). Each of these techniques present their specific advantages and disadvantages. For example, optogenetic modulation relies on the use of genetically encoded non-native proteins and is hampered by a limited light penetration, which reduces the potential target brain area. One of the latest approach is based on magneto-thermal neuromodulation method involving heat-sensitive cell thermoreceptors, such as TRPA1-A (Rosenfeld et al., 2022). However, there are several questions to be addressed to promote the clinical translation of such nanotechnology, related to the local and repeated temperature increase of the brain tissue and the possible risk of off-target heating (Owen et al., 2019; Davis et al., 2020). In light of these considerations, magneto-electric neuromodulation approaches which relies only of the use of biocompatible MENPs, activated by a safe low intensity magnetic field can bring the greatest therapeutic potential in the field.

In this context, the most widely used configuration of MENPs is the core-shell system based on CoFe_2_O_4_ (cobalt ferrite, CFO, spinel structure)—BaTiO_3_ (barium titanate, BTO, perovskite structure). CFO is a hard magnetic material which shows a ferromagnetic behavior at room temperature, while BTO presents spontaneous electric polarization and piezoelectric properties. However, other materials combinations have been proposed in the literature, with Fe_3_O_4_, NiFe_2_O_4_ as cores, and PbTiO_3_ and BiFeO_3_ as shells components (Niranjan et al., 2008; Koo et al., 2009; Banerjee et al., 2018; Song et al., 2022). Core/shell nanostructures are widely adopted to maximize a good interfacial coupling between the core and the shell and, therefore, to ensure a proper mechanical-to-electrical signal transduction (Smith et al., 2022; Song et al., 2022). In this regard, tuning the electro-magnetic properties by changing the morphology of the MENPs from the traditional spherical particles to other nanostructures (e.g., nanorice, nanorods, nanotubers, nanowires, etc.) opens up enormous possibilities and widens MENPs applications and performances. In this framework, *in silico* investigations are of paramount importance to define the MENPs operational range for a specific bio-application.

Considering the above-mentioned premises, in this work, the magnetoelectrical behavior, in terms of magnetoelectric coupling coefficient (αME), of core-shell MENPs with different shapes was analyzed through a numerical model. The nanostructures were initially subjected to a high amplitude DC magnetic field to assess their electrical output. Moreover, a following study was performed under a time-variant and low amplitude magnetic field at low frequency (50 Hz), as several recent works reported how this type of magnetic stimulation commonly adopted for biomedical applications can yield to an improved magnetoelectric response (Guduru et al., 2013; Nair et al., 2013; Betal et al., 2016b; Kaushik et al., 2017; Betal et al., 2018; Fiocchi et al., 2022b). Results of this work pinpoint the possibility of efficiently modulating the MENPs electrical output also by varying their geometrical features, an approach less investigated so far. This is of primary importance when designing new-generation of nano-tools and exploring novel magnetoelectric materials configurations, where the full exploitation of their potentialities in a specific bio-application is strongly linked to their shapes and sizes.

## 2 Methods

### 2.1 Nanoparticles modeling

COMSOL Multiphysics^®^ 5.6 (www.comsol.com) was adopted to model the MENPs magnetoelectric behavior by tuning their geometrical features. They were modelled as core-shell structures by using an axisymmetric bi-dimensional (2D) model. In particular, all MENPs are composed of a piezoelectric shell (BTO) and a magnetostrictive core (CFO). Material properties of the CFO core and the BTO shell were found in the literature (Betal et al., 2016b; Chinnasamy et al., 2003; García Saggión et al., 2020; Kumar et al., 2021; Kurian et al., 2015; X; Zhao et al., 2018) and in the COMSOL built-in library (www.comsol.com), as reported in Supplementary Table S1, S2.

**FIGURE 1:**
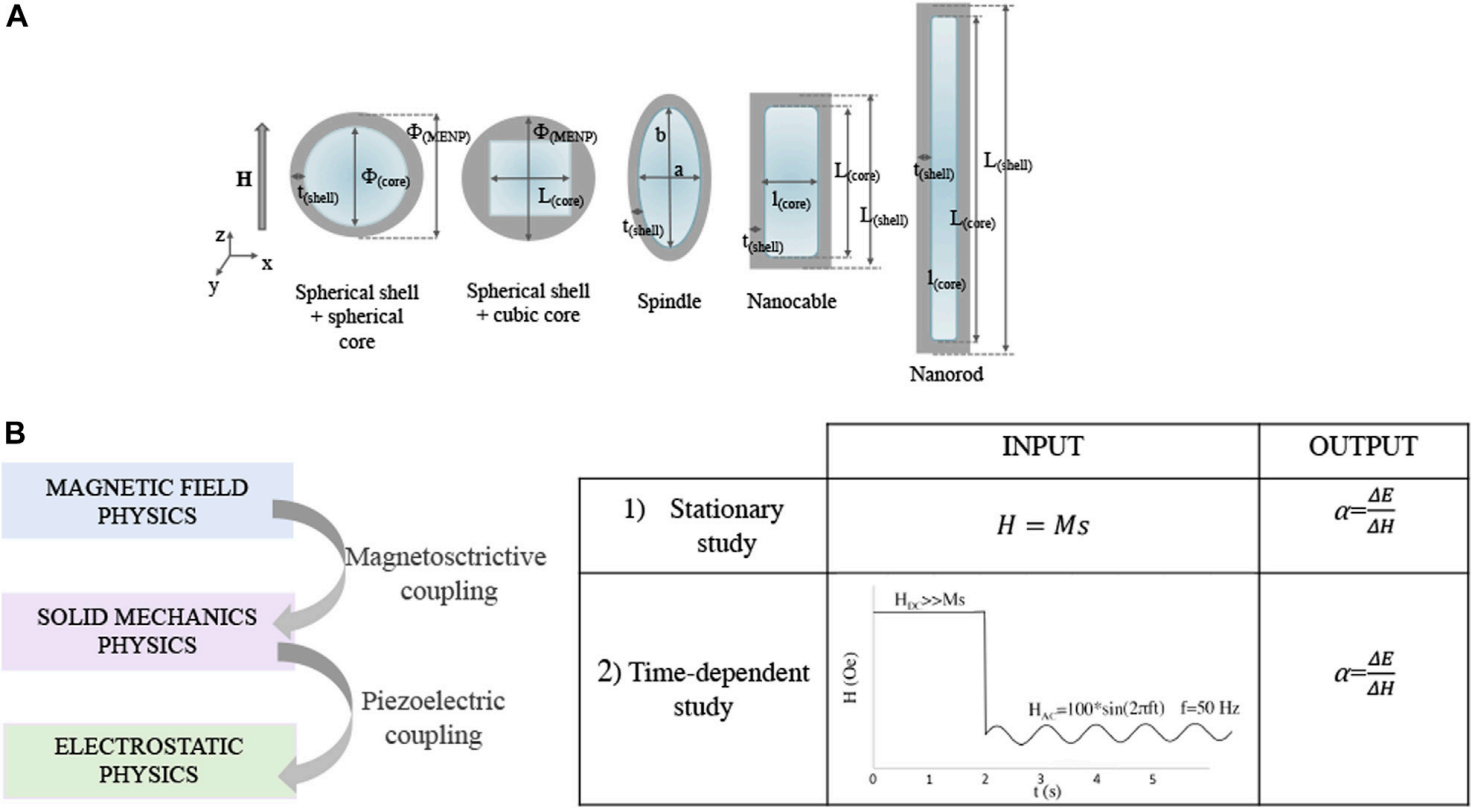
MENPs morphologies and modeling parameters. **(A)** Sketches representing the different magnetoelectric nanostructures under study, with the corresponding dimensions. **(B)** MENPs computational study workflow and simulation settings.

Different morphologies were considered in this study, as shown in Figure 1: sphere with spherical core (SPH), sphere with cubic core (SPH-C), spindle (SPI), nanocable (NCB), nanorod (NR). The MENPs diameters, core diameters and shell thicknesses are reported in Figure 1A and Table 1.

**TABLE 1:** MENPs morphological parameters.

MENP shape	Label	Dimensions
Spherical shell + Spherical core	SPH	Ф_(MENP)_ = 190 nm; Ф_(core)_ = 150 nm; t_(shell)_ = 20 nm
Spherical shell + Cubic core	SPH-C	Ф_(MENP)_ = 190 nm; L_(core)_ = 100 nm
Spindle	SPI	Semiaxis a _(core)_ = 40 nm; Semiaxis b _(core)_ = 75 nm, t_(shell)_ = 20 nm
Nanocable	NCB	L_(core)_ = 140 nm; l_(core)_ = 100 nm; L_(shell)_ = 180 nm; t_(shell)_ = 20 nm
Nanorod	NR	L_(core)_ = 360 nm; l_(core)_ = 40 nm; L_(shell)_ = 400 nm; t_(shell)_ = 20 nm

The first geometries designed (i.e., SPH, SPI, NCB) presented comparable volumes (Figure 1). For the case of the NR, two main constraints have been considered: a) its morphology implies a strong preferential axis of growth; b) the core volume has to be comparable with those of the other structures, since this feature strongly affects the electrical performance (Fiocchi et al., 2022b). Therefore, the NR dimensions were set in order to 1) keep rather constant the core volume with respect to the other geometrical configurations, 2) guarantee a strong geometrical anisotropy (>2), and 3) being consistent with NRs fabricated in experimental studies (García Saggión et al., 2020; S; Mushtaq et al., 2022; Raidongia et al., 2010; Si et al., 2014).

Firstly, stationary studies were performed to assess the influence of the MENPs geometry on the magnetoelectric coefficient applying an external magnetic field above the magnetic saturation (Ms), directed along the *z*-axis. Then, time-dependent analyses were run to characterize the MENPs performance dependence on the geometry, when an AC magnetic field directed along the *z*-axis at low frequency and low amplitude (f = 50 Hz, 100 Oe) is applied after a DC pre-magnetization through high amplitude excitation (>> Ms) (Figure 1B). This second set of analyses was carried out for the MENPs nanostructures presenting a constant shell thickness (i.e., SPH, SPI, NCB, and NR), in order to better appreciate the impact of the particle shape on its electrical output. The mechanical boundary condition was set in the center of the CFO core and the electrical ground was applied along the *x*-axis at a distance of 1,000 nm from the center of the MENP. Influences from the surrounding environment (medium) on the shell electrical surface potential were neglected (the relative permittivity and permeability are assumed to be 1).

For all the analyses, the MENPs performance was assessed through the calculation of the magnetoelectric coefficient, which is the ratio between the maximum E field intensity, derived from COMSOL modeling, at the MENPs outer border and the change in external magnetic field H:

$$\alpha_{ME}=\Delta E/\Delta H$$ (1a)

For all the configurations, the interface area between the CFO core and BTO shell as well as the geometrical anisotropy (i.e., the axes ratio) were derived.

### 2.2 COMSOL multiphysics model

Magnetoelectric behavior is modelled by coupling the *Magnetic Fields*, *Solid Mechanics*, and *Electrostatics* COMSOL Modules, as schematically resumed in Figure 1B. The *Multiphysics* mode was employed through the coupled *Magnetostriction* and *Piezoelectric Multiphysics*. MENPs were defined as a bulk material placed in medium. The model was composed by three subdomains: 1) the magnetostrictive core; 2) a piezoelectric phase and 3) medium. The *Magnetostatics* mode was active in all domains. Details about the mathematical equations governing the model are derived from Fiocchi et al. (2022b) and reported in SI. On the other hand, having modified the hysteretic modelling based on the different MENPs geometries, details about the related theoretical framework are herein presented.

The magnetic hysteresis is modelled through the Jiles–Atherton (J–A) model (Pop and Caltun, 2010; 2011; Pop and Călţun, 2012), which is based upon the following parameters: the magnetization reversibility (c), the saturation magnetization (Ms), the domain wall density (a), the pinning loss (k), and the inter-domain coupling (α). This model is based on two main hypotheses: 1) each domain magnetic moment is subjected to an external magnetic field which linearly increases with the magnetization of the material, 2) several impurities, called pinning sites, are homogenously distributed within the material.

In particular, the key equation in the Jiles-Atherton model describes the variations in the total magnetization **M** due to the changes of the effective magnetic field as follows:

$$\frac{dM}{dt}=\max\left(\chi \;dHeff,0\right)\frac{\chi }{\left|\chi \right|}+c\frac{dM_{an}}{dt}$$ (1b)

where the auxiliary vector χ is defined as:

$$\chi =\frac{M_{an}-M}{k}$$ (2)

Where $M_{an}$ is anhysteretic magnetization, which is defined by Equation 7.

The effective magnetic field **H*eff*** is described by the following equation where the inter-domain coupling parameter (α) is a measure of the coupling between the adjacent magnetic domains*:*

$$H_{eff}=H+\alpha M$$ (3)

The total magnetization of a material (***M***) has both a reversible (***M***_***rev***_) and an irreversible $\left(M_{irr}\right)$ component, due to the elastic bending of magnetic domain wall and the discontinuities in the material structure, respectively:

M=Mrev+Mirr (4)

The irreversible component of the magnetization is given by the following differential equation:

$$\frac{dM_{irr}}{dt}=g\left(\frac{M_{rev}}{c\;k}∙\frac{dH_{e}}{dt}\right)\frac{M_{rev}}{\left|M_{rev}\right|}$$ (5)

Where*, k* is the pinning loss, *c* is a measure of the magnetization reversibility and g=1 if dH/dt>0 and g=−1 if dH/dt<0 .

The reversible part of magnetization can be expressed with the following equation:

$$M_{rev}=c\left(M_{an}-M_{irr}\right)$$ (6)

where the reversible magnetization represents a certain percentage “c” of the difference between the anhysteretic magnetization (***M***_***an***_) and the irreversible one.

***M***_***an***_ is described the Langevin’s function, as follows:

$$M_{an}=M_{s}\left(\coth\frac{\left|H_{eff}\right|}{a}-\frac{a}{\left|H_{eff}\right|}\right)\frac{H_{eff}}{\left|{H_{eff}}_{e}\right|}$$ (7)

where *a* is proportional to the magnetic domain density, and *M*_*s*_ is the saturation magnetization.

**H*eff*** is the effective magnetic field inside the magnetostrictive core and it is described by the following equation:

$$Heff=H+\alpha M+\frac{3\lambda_{s}}{\mu_{0}M_{s}^{2}}S_{dev}M$$ (8)

where S_dev_ is the deviatoric part of the stress tensor, which is computed in the *Solid Mechanics* module. The last term is known as “Villari effect” which represents the mechanical stress contribution to the material magnetization. This term depends on the saturation magnetization *Ms*, the magnetostriction coefficient λs, as well as the deviatoric stress tensor. To obtain the hysteresis curves for the sphere and the nanorod configurations, the COMSOL entries for the Jiles–Atherton (J–A) model parameters have been modified accordingly to Supplementary Table S3, following the theoretical work conducted by Pop and Caltun, (2010), Pop and Caltun, (2011); Pop and Călţun, (2012). The output curves were analyzed via OriginPro 2022 (OriginLab, United States) to extrapolate the values of *H*_*c*_ (coercitivity field) and *M*_*r*_ (magnetic remanence).

## 3 Results and discussion

Nanoparticle shape and anisotropy act as key parameters in nanomaterials-cellular interaction processes both at the cellular membrane level and when internalized within the cytosolic compartment (Jindal, 2017). In particular, internalization mechanisms, uptake kinetics and intracellular fate are dictated by nanoparticle morphology and dimensions (Suarato et al., 2016; Yang et al., 2019). Several experimental studies have reported that nanostructures with high aspect ratios elicit an efficient cellular uptake, a faster internalization rate, an increased ability to escape endosomal digestion and an overall greater impact on several cellular functions (i.e., proliferation, apoptosis, adhesion, and migration) (Huang et al., 2010; Meng et al., 2011; Barua et al., 2013; Chu et al., 2014; Yang et al., 2016; Mushtaq et al., 2022). Moreover, it has been shown that non-spherical nanoparticles are characterized by a prolonged circulation time, a favorable biodistribution and a consequent substantial tissue penetration (Zhang et al., 2014).

In light of these experimental evidences in our modeling framework different shapes have been modelled starting from the sphere as reference structure upon which various geometries have been constructed with similar volumes but different anisotropy. The reference core-shell nanosphere has been modelled based on the results of a preliminary study conducted by our group (Fiocchi et al., 2022b), where a maximized α_ME_ was obtained when a spherical core diameter of 150 nm and a shell thickness of 20 nm were coupled. While keeping constant the thickness of the outer component, various geometries of increasing anisotropy have been considered (SPI, NCB, NR), to better assess the effect of the nanoparticle shape on the electrical response.

**FIGURE 2:**
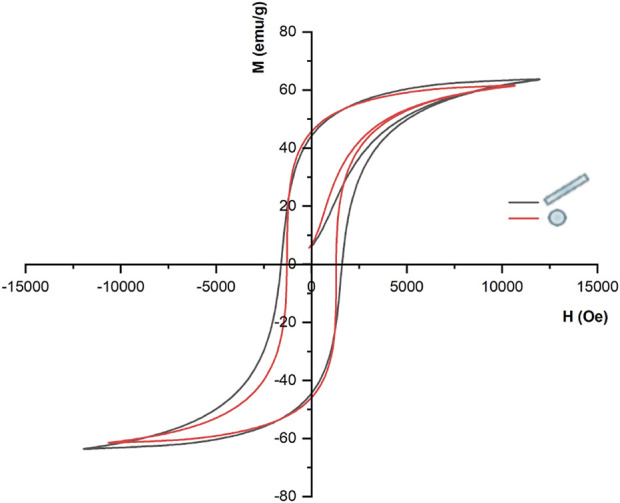
Hysteresis curves for the sphere and the nanorod structures.

Firstly, we performed iterative simulations to find the Jiles-Atherton model input parameters to obtain hysteretic theoretical curves that best replicate the magnetization curves experimentally measured for the case of nanosphere and nanorod with sizes comparable with our constructs. It has been reported that the saturation magnetization (Ms) and coercivity field (Hc) values are strongly dependent on the particle size (Chinnasamy et al., 2003; Raidongia et al., 2010; Das et al., 2016; García Saggión et al., 2020). More specifically, experimental studies indicated that for the NR, whose geometrical architecture presents a marked anisotropy, Hc and Ms values should be higher than those of the SPH (Antonel et al., 2015; Ghaemi et al., 2021). Therefore, in the present model a Ms of 3.9 × 10^5^ A/m was chosen for the NR particle, accordingly to Raidongia et al. (2010), and a Hc of 1,608 Oe was calculated from the obtained graph (Figure 2, black loop). For the SPH, Ms was set as 3.69 × 10^5^ following Chinnasamy and co-workers (Chinnasamy et al., 2003), while an Hc of 1,293 Oe was derived from the theoretical curve (Figure 2, red loop). When grain boundaries are present within the core material (a configuration far from the “single-domain regime”), the interactions among the various magnetic grains are strong. As a result, the magnetization reversal is guided by the process of “disclination motion” of domains, which requires a higher coercivity field. This mechanism is emphasized in narrower structures, such as nanorods and nanotubes (Antonel et al., 2015; García Saggión et al., 2020). Considering these premises, in our study the SPI and NCB nanoparticles have been modelled as the SPH from the hysteric point of view, while for the NR nanoparticles different parameters were considered (Supplementary Table S3).

**FIGURE 3:**
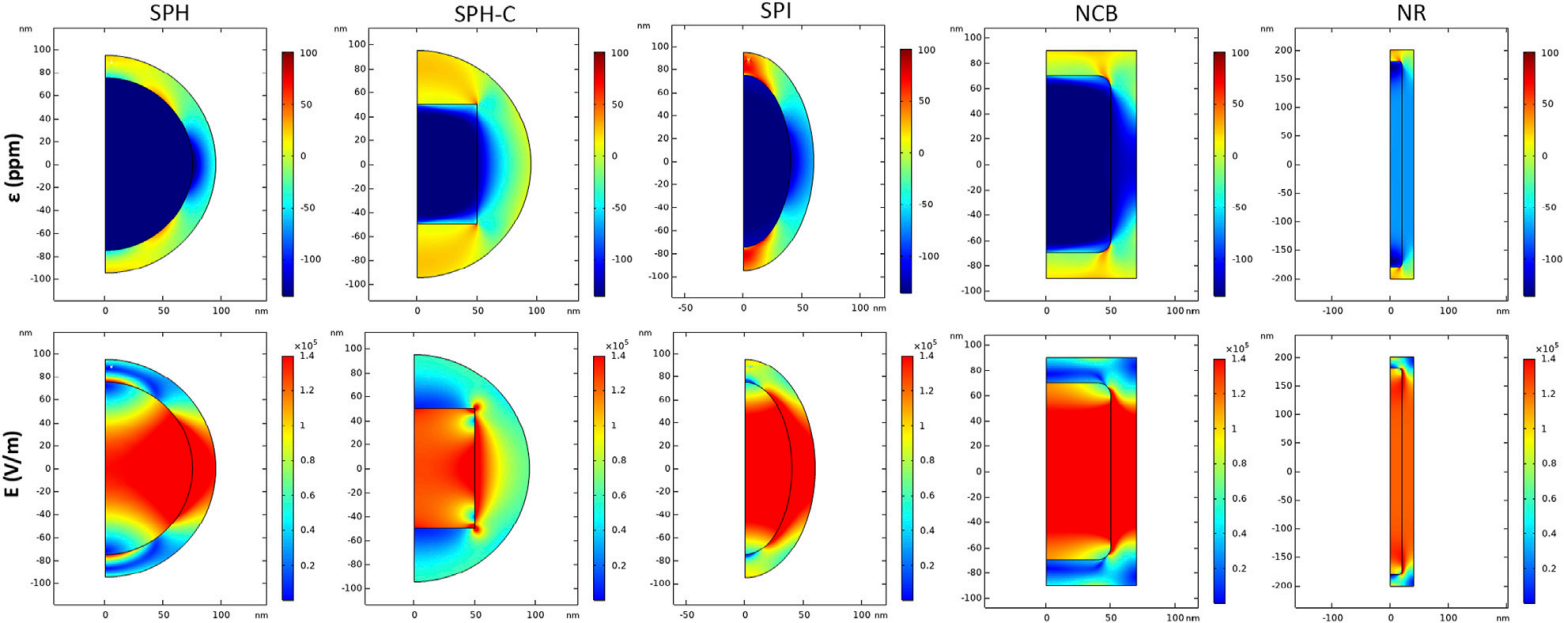
Magnetoelectric effect induced by DC magnetic field stimulation. Distribution of strain ε (ppm) and electric field E (V/m) in 2D axisymmetric CFO-BTO core-shell nanostructures, when a DC magnetic field at saturation is applied in the study (H = Ms).

**FIGURE 4:**
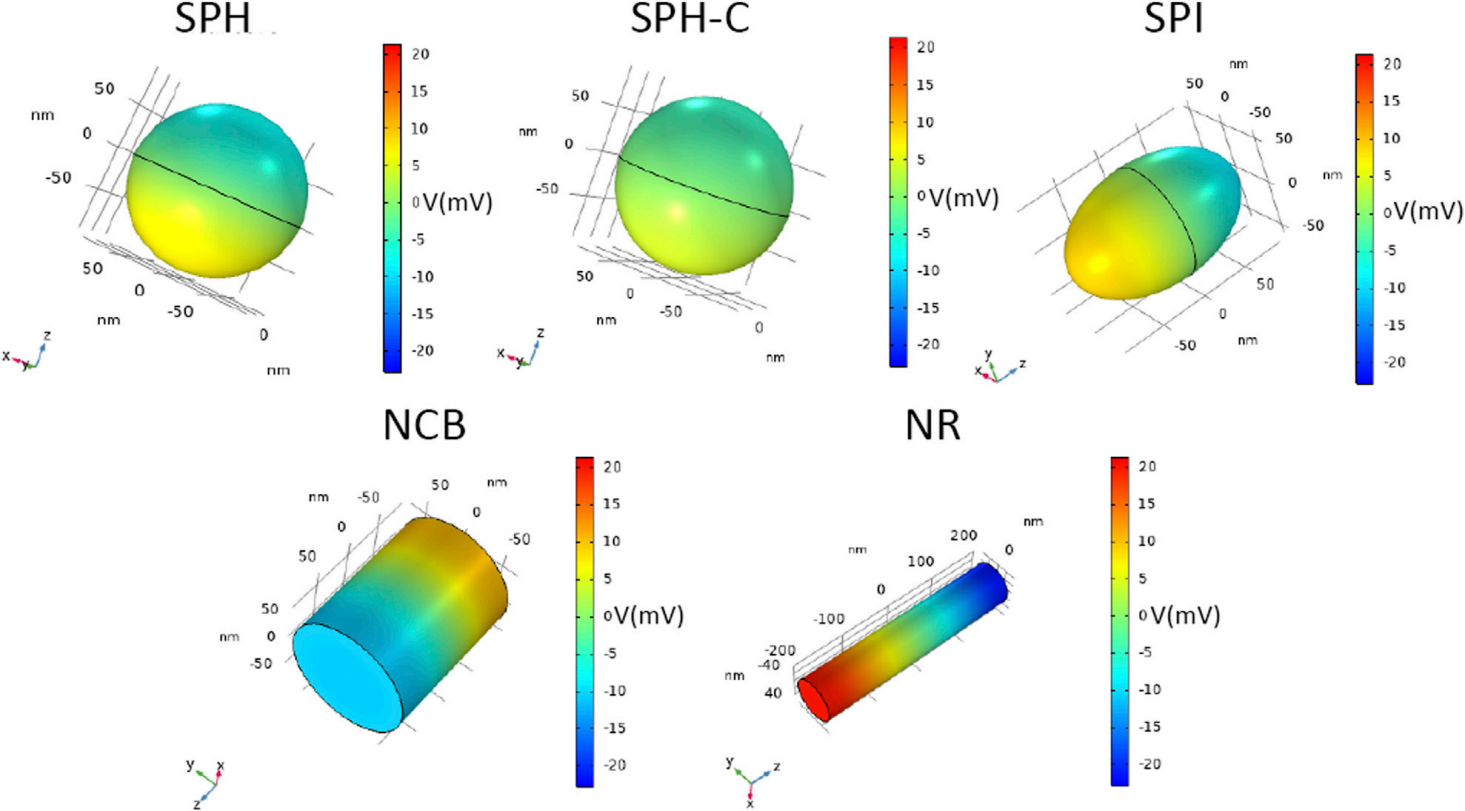
Magnetoelectric effect induced by DC magnetic field stimulation. 3D distribution of electric potential V (mV), of CFO-BTO core-shell nanostructures, when a DC magnetic field at saturation is applied in the study (H = Ms).

Figure 3 quantifies the core magnetostriction behavior and the related electric field module when a strong DC bias magnetic field (H = Ms) is applied. During the initial magnetization step, the core experiences a change in its dimension and a compressive strain along the z-axis. This strain then propagates to the piezoelectric shell, which converts the consequent mechanical stress to a change in the electric field distribution (Figure 3, bottom row) and surface potential (Figure 4). From the simulation results, it is visible that the strain is transferred through the core-shell interface in all cases, but it propagates more homogeneously in the anisotropic structures (NR > NCB > SPI > SPH). For the SPH the strain variation resulting in the BTO shell is more localized. On the contrary, for what concerns the NR, a maximum strain is obtained around the edges of the core, while the inner region is subjected to an overall lower deformation, which nonetheless is more homogenously transferred to the external piezoelectric phase. This is of primary importance to elicit a valuable electrical response. In fact, the following maximum values of E (V/m) are obtained: 1.36 × 10^5^ for the SPH, 0.66 × 10^5^ for the SPH-C, 1.93 × 10^5^ for the SPI, 3.67 × 10^5^ for the NCB, and 7.40 × 10^5^ for the NR.

As strikingly visible in Figure 4, the electric potential difference generated between the extremities of the nanostructures increases by changing their shape (from the spherical reference to more elongated structures), reaching a maximum value of 20.5 mV for the NR, thus suggesting the most efficient magnetoelectric behavior.

The SPH-C resulted in a lower electric field propagation, if compared with the SPH E profile, most likely because of a less geometrically compliant interface between the magnetostrictive and the piezoelectric phases, in combination with an inhomogeneous, higher thickness of the BTO shell (Fiocchi et al., 2022a). For this reason, this modelled construct was not evaluated in the following analyses.

**FIGURE 5:**
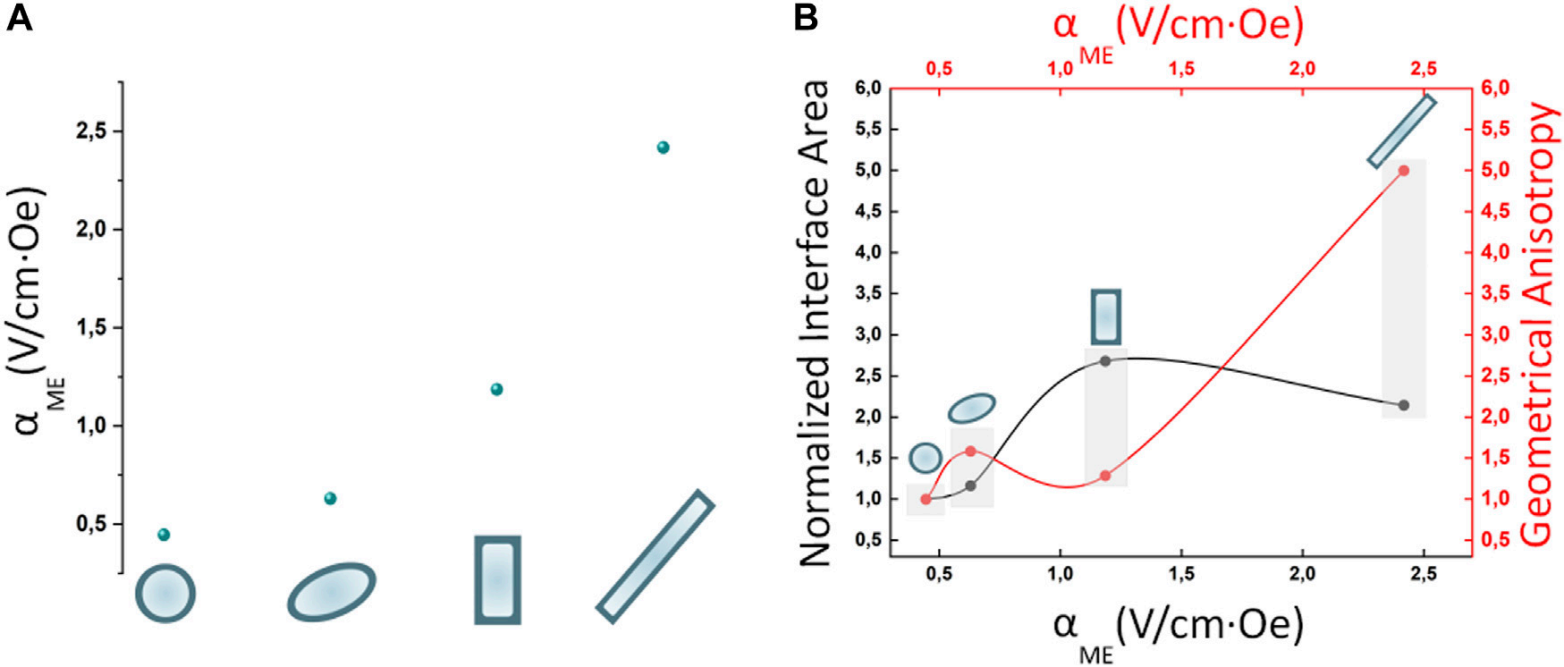
Magnetoelectric nanostructures performances. **(A)** Effect of the nanostructures geometries on the magnetoelectric coeffient α_ME_ (V/cm*Oe) when MENPs are stimulated at saturation (H = Ms) in DC conditions. **(B)** Influence of normalized interface area and geometrical anisotropy on the magnetoelectric performance. For all the configurations under study the MENPs are oriented along the direction of the applied external magnetic field H.

The magnetoelectric coefficient was calculated as the ratio between the maximum E field intensity derived at the MENPs outer border and the change in the external magnetic field H (Table 2; Figure 5). The electric performance remarkably improved as follows (NR >> NCB > SPI > SPH). The area at the interface between the magnetostrictive and piezoelectric phase along with the geometrical anisotropy of the structures under study were calculated (Table 2). As shown in Figure 5 and resumed in Table 2, the NCB and NR interfacial areas are comparable and higher than the SPH and SPI systems, while the anisotropy is incremented of 5-folds when passing from the nanosphere to the nanorod. Taken together, these observations suggest that the interplay between the contributions of both the interface coupling and the shape anisotropy is essential to achieve an ameliorated magnetoelectric response. This is consistent with what reported in some experimental studies that showed how the ME properties could be modulated through a proper interface engineering (Betal et al., 2016a; Elissalde et al., 2018). In fact, when the magnetostrictive and the piezoelectric phases are tightly bonded, the strain generated in the core homogenously transfers to the shell, thus more efficiently triggering the electrical field generation. In parallel, the anisotropy of the composite nanostructure fundamentally influences the MENPs magnetoelectrical behavior. This is in accordance with experimental measurements involving structures with even a more pronounced aspect ratio (e.g., nanowires, nanofibers), whose electrical response is highly dependent on their orientation with respect to the external magnetic field direction (Biswas et al., 2017). It is worth noticing that obtaining nanoparticles characterized by a marked geometrical anisotropy or by a controlled morphology is more cumbersome than the synthesis of simple, spherical systems and it may require subsequent steps and the use of nanosized templates such as anodized aluminum oxide (Biswas et al., 2017; Chen et al., 2017; Elissalde et al., 2018), polymeric precursors (Baji et al., 2014) or various surfactants (Chen et al., 2005; Bao et al., 2012; Chen et al., 2017). However, the fabrication of either magnetite-based or core-shell MENPs of various geometries has been widely reported in the literature, suggesting the feasibility of these different techniques (Chen et al., 2005; Bai et al., 2010; Sun et al., 2012; Si et al., 2014; Antonel et al., 2015; Das et al., 2016; Xu et al., 2019; Zhao et al., 2021). In this context, the use of *in silico* modeling to predict the behavior of specific and unexplored geometries allows to select the best configurations before embarking on their synthesis.

**TABLE 2:** Magnetoelectric nanostructures geometrical features and performances.

Geometry	Interface area (nm^2^)	Normalized interface area	Geometrical anisotropy	α_ME_ (V/cm*Oe) @ H = M_s_
Spherical core—SPH	2.83*10^4^	1.00	1.00	0.44
Spindle—SPI	3.30*10^4^	1.16	1.58	0.63
Nanocable—NCB	7.60*10^4^	2.68	1.29	1.19
Nanorod—NR	6.08*10^4^	2.15	5	2.40

Low-frequency time-variant electric field can be exploited to interact with biological systems: electric field pulses can be adopted to boost the intracellular delivery of chemicals and genes (Choi et al., 2014; Marrella et al., 2018), and to induce cellular differentiation and tissue growth (Mushtaq et al., 2019; Kopyl et al., 2021). In particular, when MENPs are subjected to a time-variant magnetic field (AC) a vibrational lattice strain in the magnetostrictive core occurs, which is then transferred to the piezoelectric shell inducing an alternating electrical polarization. In drug delivery applications, the generation of an oscillating dipole allows the weakening of the physical bonds between the therapeutic molecules and the piezoelectric shell, thus promoting the drug release in the surroundings of the diseased tissue/organ. A step forward in the targeting delivery is constituted by the nano-electroporation approach, which takes advantage of the alternating electric field generated at the piezoelectric outer region to modify the transmembrane potential, dislocate the phospholipidic bilayer and temporarily porate the cell membrane, thereby facilitating the drug loaded-MENPs entrance in the cytosolic compartment. For example, Kaushik et al. (2017) explored this approach to deliver MENPs to brain cells *in vitro* under an AC magnetic field of 40–60 Oe at a frequency of 1 kHz and study their enhanced uptake and intracellular diffusion profile, which did not correlate with cell toxicity nor with local intense heat production.

**FIGURE 6:**
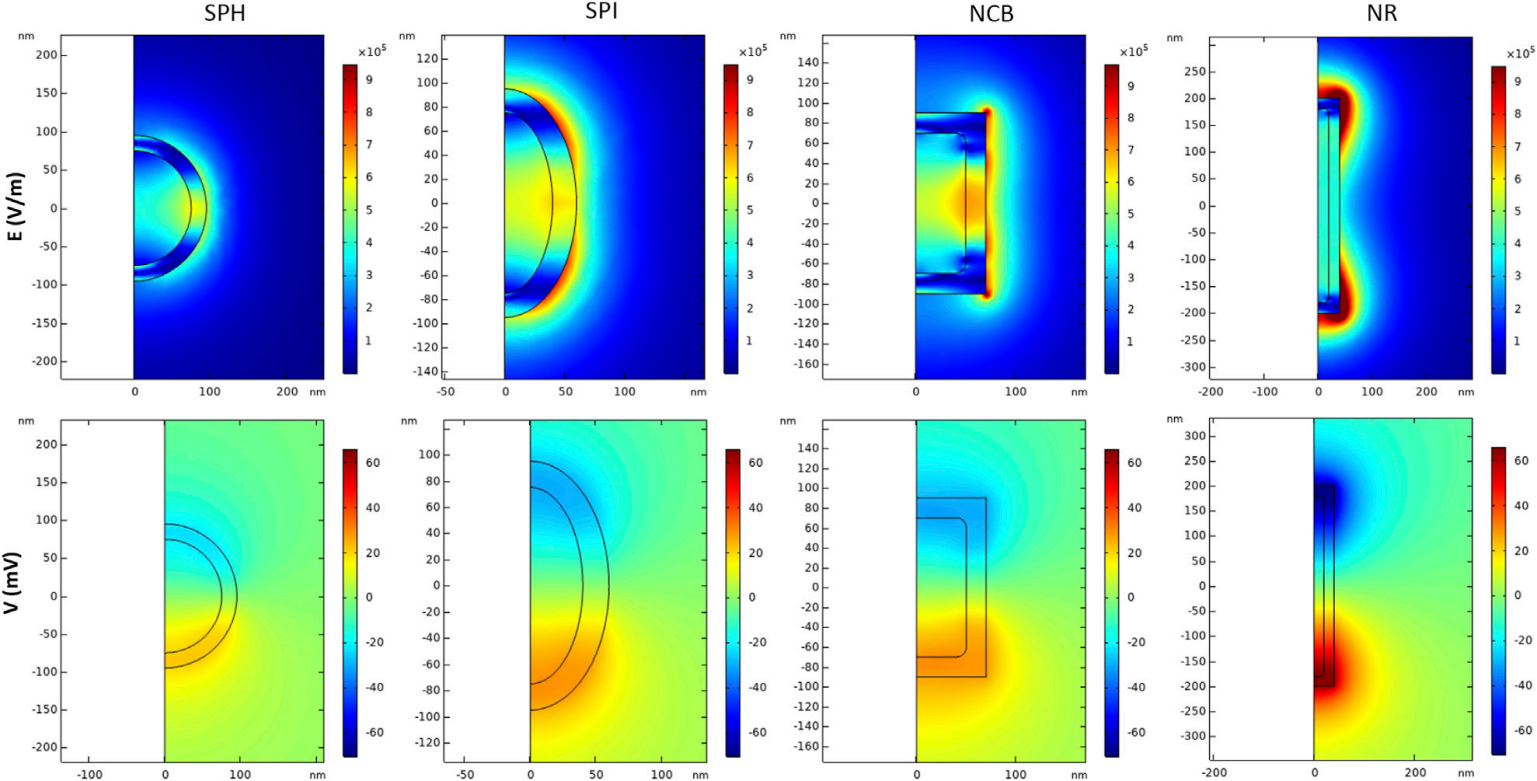
Magnetoelectric effect induced by AC/DC magnetic field simulation. Distribution of electric field E (V/m) and electric potential V (mV) in 2D axisymmetric CFO-BTO core-shell nanostructures, when a high amplitude DC magnetic field (H > M_s_) + low amplitude AC bias magnetic field are applied in the study. Data are reported for t = 4 s.

Figure 6 shows the electric field module and potential when a time-variant external magnetic field composed by a high DC bias (>> Ms) is applied for the first 2 s of stimulation, followed by a lower amplitude sinusoidal magnetic field excitation (f = 50 Hz, 100 Oe) up to 5 s. According to our results, the SPH nanoparticle generates (at 4 s of stimulation, which stands within the AC magnetic field regime) a maximum electric field value of 5.7 × 10^5^ V/m when only the 100 Oe AC field is applied, corresponding to a magnetoelectric coefficient of above hundreds of V/cm*Oe. On the other hand, the NR configuration generates a maximum local electric field of 1.1 × 10^6^ V/m that can reach up values above two hundreds of V/cm*Oe. Therefore, the same trend observed in static conditions is maintained by applying an alternating magnetic field. In addition, it is visible from our modeling results (Figure 6) that the electric field reaches higher values around the edges of the sharp nanostructures (i.e., NCB and NR). This analysis confirms that with a low amplitude AC magnetic field the ME effect can be drastically enhanced, if the core has been pre-magnetized with a high DC field, as already proved by a previous work (Fiocchi et al., 2022a). This is of primary importance when dealing with biomedical applications at the clinical stage because it allows to expose human tissues to lower, thus safer, external magnetic fields to activate the MENPs.

## 4 Conclusion

Magnetoelectric nanoparticles constitute a cutting-edge strategy in biomedicine and nano-theranostics by steering a wireless route to human tissues. Starting from the well-established MENPs with a spherical morphology, in this study we investigated how other shapes can be of advantage to enhance the magnetoelectric response. Our results show that more elongated core-shell structures generate a higher ME output, thanks to the interplay between an optimized interface coupling and a more pronounced shape anisotropy in the direction of the applied magnetic fields. This trend is observed both under DC-bias and low-frequency AC-bias magnetic stimulation. Moreover, the locally induced electrical field becomes drastically enhanced under the low amplitude AC field if the material has been pre-magnetized, leveraging on the “memory effect” of the core hysteretic behavior. The herein presented concept can be broadened to engineer various geometrical interphases configurations, thus gaining additional knowledge to support MENPs synthesis, properly tailored for biomedical applications.

## Data Availability

The original contributions presented in the study are included in the article/Supplementary Material, further inquiries can be directed to the corresponding authors.
