# Supplementary material for: Magnetoelectric nanoparticles shape modulates their electrical output
Source: Front Bioeng Biotechnol. 2023 Aug 25;11:1219777. doi: 10.3389/fbioe.2023.1219777 (PMC10485842; doi:10.3389/fbioe.2023.1219777)
Supplement: Supplementary file 1 [file DataSheet1.docx]

Supplementary Material

Magnetoelectric nanoparticles shape modulates their electrical output

**A. Marrella^*,1,†^, G. Suarato^*,1, †^, S. Fiocchi^1^, E. Chiaramello^1^, M. Bonato^1^, M. Parazzini^1^, P. Ravazzani^1^**

^1^Cnr-Istituto di Elettronica e di Ingegneria dell’Informazione e delle Telecomunicazioni, Milano, Italy

# * Correspondence: Corresponding Author [alessandra.marrella@ieiit.cnr.it](mailto:alessandra.marrella@ieiit.cnr.it) , [giulia.suarato@ieiit.cnr.it](mailto:giulia.suarato@ieiit.cnr.it)

**^†^** Co-first authors

**COMSOL Multiphysics models**

**Magnetic Field Module**

For what concerns the Magnetostatics mode the relationship of the magnetic flux density (B) versus the magnetic field H (letters in bold represent vectors) can be expressed as the equations (1) and (2):

$\boldsymbol{B}=\boldsymbol{\mu}_{\mathbf{0}}\boldsymbol{\mu}_{\boldsymbol{r}}\boldsymbol{H}$ (1)

which is applicable for the piezoletric shell and the surrounding non-magnetic phases.

The following equation represents the nonlinear relationship between the magnetization (M) and H field in the magnetostrictive core (being e_H_ the unit vector along H field):

$\boldsymbol{B}=\boldsymbol{f}\left( \left| \boldsymbol{H} \right| \right)\mathbf{e}_{\mathbf{H}}$ (2)

**Solid Mechanics Module**

In the Solid mechanics module, different materials features (e.g., Young’s modulus, Poisson’s ratios, densities, etc.,) are involved in the computation. In particular, CFO core magnetostriction is governed by the following equation, which relates the magnetostrictive strain (ε_me_) of an isotropic material with its magnetic properties:

$\boldsymbol{\varepsilon}_{\boldsymbol{me}}=\frac{\mathbf{3}\boldsymbol{\lambda}_{\boldsymbol{s}}}{\boldsymbol{\mu}_{\mathbf{0}}\boldsymbol{M}_{\boldsymbol{s}}^{\mathbf{2}}}\boldsymbol{dev}\left( \boldsymbol{M}\otimes\boldsymbol{M} \right)$ (3)

For what concern the mechanical stress at the piezoelectric shell, it is defined by the following expression:

$\boldsymbol{S}=\boldsymbol{S}_{\mathbf{0}}+\boldsymbol{C}: \boldsymbol{\varepsilon}+\boldsymbol{E}\cdot\boldsymbol{e}$ (4)

where $\boldsymbol{e}$ is the piezoelectric Voigt coupling matrix representing the stress tensor, $\boldsymbol{C}$ is the elastic right Cauchy deformation tensor and E is the electric field which is computed by using the piezoelectric coupling.

Electrostatic Module

In the Electrostatic module, Gauss’ Law was solved:

$\nabla\cdot\boldsymbol{D}=\boldsymbol{\rho}_{\boldsymbol{v}}$ (5)

$\mathbf{E}=-\nabla\boldsymbol{V}$ (6)

where $\boldsymbol{D}$ is the electric flux density and $\boldsymbol{\rho}_{\boldsymbol{v}}$ is the volume charge density.

In the piezoelectric shell $\boldsymbol{D}$ is linked to the solid mechanics according to the following equation:

$\mathbf{D}=\varepsilon_{0}\mathbf{E}+\varepsilon_{0}\chi\mathbf{E}+\boldsymbol{e}: \boldsymbol{\varepsilon}$ (7)

where ε0 is the vacuum electric permittivity and χ is the relative electrical susceptibility.

**Supplementary Table 1.** MENPs core properties

|  | **Symbol** | **Value** | **Reference** |
| --- | --- | --- | --- |
| Magnetic Saturation (A/m) | M_S_ | 3.69*10^5^ | (Chinnasamy et al., 2003) |
| Magnetic Saturation (A/m) - NR | M_S_ | 3.9*10^5^ | (García Saggión et al., 2020) |
| Saturation magnetostriction (ppm) | λ_S_ | -200 | (Betal et al., 2016; Zhao et al., 2018) |
| Density (kg/m^3^) | ρ | 5200 | (Kurian et al., 2015), COMSOL Library |
| Initial magnetic susceptibility | χ_0_ | 200 | (Betal et al., 2016), COMSOL Library |
| Electrical conductivity (S/m) | σ | 5.2*10^6^ | COMSOL Library |
| Poisson ratio | ν | 0.48 | (Zhao et al., 2018), COMSOL Library |
| Young’s modulus (GPa) | E | 230 | (Kumar et al., 2021; Zhao et al., 2018) |
| Relative permeability | μ_r_ | 1 | COMSOL Library |

**Supplementary Table 2.** MENPs shell properties (from COMSOL library)

|  | **Symbol** | **Value** |
| --- | --- | --- |
| Density (kg/m^3^) | ρ | 5700 |
| Relative permittivity | {εr_11_; εr_22_; εr_33_} | {1115.1;1115.1; 1251.3} |
| Electrical conductivity (S/m) | σ | 178.5 |
| Elasticity matrix, Voigt notation  (GPa) | {cE11; cE12; cE22; cE13; cE23; cE33; cE14; cE24; cE34; cE44; cE15; cE25; cE35; cE45; cE55; cE16; cE26; cE36; cE46; cE56; cE66} | {150.4;65.6;150.4;65.; 65.9; 145.5; 0; 0;0;43.9;0; 0; 0;  0;43.9; 0; 0; 0; 0; 0; 42.4} |
| Piezoelectric coupling matrix,  Voigt notation (C/m^2^) | {eES11; eES21; eES31; eES12; eES22; eES32; eES12; eES23; eES33; eES14; eES24; eES34; eES15; eES25; eES35; eES16; eES26; eES36} | {0; 0; -4.32; 0; 0; -4.32;  0; 0; 17.4; 0; 11.4; 0;  11.4; 0; 0; 0; 0; 0} |

**Supplementary Table 3.** Jiles-Atherton model parameters for magnetic hysteresis

|  | **Symbol** | **Sphere** | **Nanorod** |
| --- | --- | --- | --- |
| Magnetic Saturation (A/m) | M_S_ | 3.69*10^5^ | 3.9*10^5^ |
| Pinning loss (A/m) | k | 2 | 2.5 |
| Domain wall density (A/m) | a | 1.5 | 1.8 |
| Inter-coupling domain | α | 1.4 | 1.4 |
| Magnetic reversibility | c | 0.4 | 0.4 |
